# Radiographic progression can still occur in individual patients with low or moderate disease activity in the current treat-to-target paradigm: real-world data from the Dutch Rheumatoid Arthritis Monitoring (DREAM) registry

**DOI:** 10.1186/s13075-019-2030-8

**Published:** 2019-11-12

**Authors:** Peter M. ten Klooster, Letty G. A. Versteeg, Martijn A. H. Oude Voshaar, Inmaculada de la Torre, Francesco De Leonardis, Walid Fakhouri, Liliana Zaremba-Pechmann, Mart van de Laar

**Affiliations:** 1 Transparency in Healthcare, Hengelo, the Netherlands; 2 Arthritis Centre Twente, University of Twente, Enschede, the Netherlands; 3 Department of Psychology, Health & Technology, University of Twente, PO BOX 217, 7500 AE Enschede, the Netherlands; 4 Arthritis Centre Twente, Medisch Spectrum Twente, Enschede, the Netherlands; 5 Eli Lilly and Company, Indianapolis, IN USA; 6 Eli Lilly and Company, Windlesham, Surrey, UK

**Keywords:** Rheumatoid arthritis, Treat-to-target, Disease activity, Radiographic damage, Real-world data

## Abstract

**Background:**

The aim of this retrospective study was to examine the longitudinal association between disease activity and radiographic damage in a cohort of patients with early RA (symptom onset < 1 year) treated according to treat-to-target (T2T) therapy.

**Methods:**

Baseline to 3-year follow-up data were used from patients included in the DREAM remission induction cohort. Patients received protocolized T2T treatment, aimed at 28-joint disease activity score-erythrocyte sedimentation rate (DAS28-ESR) remission. Disease activity (DAS28-ESR and C-reactive protein, CRP) were assessed at least every 3 months; X-rays of the hand and feet at inclusion, 6 months, and 1, 2, and 3 years were scored using modified Sharp/van der Heijde scoring (SHS). Between and within-person associations between time-integrated disease activity and radiographic progression over time were examined.

**Results:**

A subset of 229 out of 534 included patients were available for analysis. At the between-patient level, time-integrated DAS28-ESR scores were not significantly correlated with progression at the 6 month and 2-year follow-up and only weakly at the 1-year (Pearson’s correlation coefficient *r* = 0.17, *P* < 0.05) and 3-year follow-up (*r* = 0.21, *P* < 0.05). Individual slopes of the relationship between DAS28-ESR and progression scores in each time interval were significantly correlated over time and the slope of the first 6 months was moderately associated with this slope at later time points (*r* between 0.39 and 0.59; *P* values < 0.001). Between 15.9 to 22.7% and 16.7 to 38.5% of patients with low and moderate time-integrated disease activity, respectively, experienced relevant (ΔSHS ≥ 3) radiographic progression at the different time intervals. Analyses using CRP showed similar results.

**Conclusions:**

In early RA patients treated according to T2T, radiographic progression appears to be an individually determined disease process, driven by factors other than consistent high disease activity. For individual patients, the intra-patient relation between disease activity and cumulative radiographic damage during the first 6 months is a good indicator for this relation in later years.

**Trial registration:**

Netherlands Trial Register NTR578, 12 January 2006.

## Background

Rheumatoid arthritis (RA) is a chronic inflammatory joint disease with a variable clinical course and a distinctive pattern of joint damage. There is robust evidence from clinical trials that ongoing disease activity in RA, as reflected by elevated disease activity parameters such as C-reactive protein (CRP) levels or composite disease activity scores, leads to more rapid joint destruction [1, 2]. Because disease activity varies over time, time-integrated—or area under the curve (AUC)—methods are best suited for summarizing the course of disease activity and facilitate comparison with outcome measures that are cumulative, such as radiographic progression [3, 4]. Several earlier studies have indeed shown that time-integrated disease activity was strongly associated with radiographic progression in patients with early RA treated with conventional synthetic disease-modifying antirheumatic drugs (csDMARDs) and/or non-steroidal anti-inflammatory drugs (NSAIDs) [5–10].

More recently, however, studies have reported considerably weaker associations or even a disconnection between inflammation and joint damage in RA patients treated with newer treatment algorithms and medications, such as in early RA patients treated with aggressive csDMARD therapy [11] and in established RA patients treated with biological (combination) therapy [12, 13]. This was supported by a recent study by Knevel et al. [14] who examined the proportion of variance in radiographic progression explained by cumulative measures of disease activity separately for three increasingly aggressive csDMARD strategies that evolved over time. This study showed that the explained variance of CRP and swollen joint counts (SJC) gradually decreased from 25 and 24%, respectively, in patients initially treated with NSAIDs, to 17 and 4% in patients initially treated with mild csDMARD therapy and to 9% and 0% in patients with early aggressive DMARD treatment.

Current treat-to-target (T2T) strategies, which focus on early and rapid reduction of disease activity, have been shown to substantially reduce radiographic damage at the group level [15, 16]. However, despite this intensive treatment, radiographic progression still occurs in a proportion of patients and early joint damage appears to predict long-term radiographic progression [17]. Taken together, these findings indicate that—especially in the current T2T paradigm—joint damage may be to a considerable extent driven by factors other than (consistent) high disease activity, and instead may be an individually determined disease process. This would mean that the relation between time-integrated disease activity and radiographic damage is different for individual patients, where there may be patients with high disease activity over time without structural damage, but also patients with limited or low disease activity over time who do show relevant radiographic progression. If so, absolute cutoffs for low disease activity or remission, such as those defined for the 28-joint disease activity score-erythrocyte sedimentation rate (DAS28-ESR) [18, 19], may not be adequate anymore for assessing the cumulative risk of long-term radiographic damage in early RA patients receiving T2T treatment, and the integrated individual time-related disease process could be the key driver of structural damage.

The exact relationship between disease activity and radiographic progression in the current era of early and intensive T2T is, however, not yet clear. Although T2T results in acceptable control of disease activity for most patients, this may not always be a benign status in relation to longer term outcomes. More knowledge about this association and the consequences for individual patients is important for optimizing treatment of early RA in daily clinical practice. Therefore, the aim of this retrospective study was to examine the longitudinal association between disease activity and radiographic progression in a real-world inception cohort of consecutive patients with early RA treated to the target of remission according to a protocolized step-up T2T strategy.

## Methods

### Data selection and study design

The Dutch Rheumatoid Arthritis Monitoring (DREAM) remission induction cohort is a multicenter observational cohort study of early RA patients treated to the target of remission [20]. Patient enrollment in the cohort took place between 2006 and 2012 and follow-up data collection is still ongoing. Adult patients with a clinical diagnosis of RA (made at the discretion of the attending rheumatologist) were included if they had a symptom duration (defined as the time from the first reported symptom to the diagnosis of RA) ≤ 1 year, had a DAS28-ESR score ≥ 2.6, and had not previously received DMARDs and/or prednisolone. Patients were included at the time of the clinical diagnosis and started T2T immediately.

For the present study, a subset of patients enrolled before 2010 at the Medisch Spectrum Twente hospital and the Isala Zwolle hospital was used, because long-term radiographic data (outcome measure) were available only in these two units [21]. Patients with at least two available radiographic assessments were selected for analysis. The Medical Ethics Committees of both hospitals determined, in accordance with Dutch Law, that no ethical approval was required for the remission induction study because all data were collected as part of regular daily clinical practice. Nevertheless, patients were fully informed and informed consent was obtained from all patients.

### Treatment

Details on the treatment protocol have been published previously [16, 20]. Briefly, patients were treated according to a T2T strategy aiming at remission (DAS28-ESR < 2.6). Patients started with initial monotherapy of 15 mg/week methotrexate (MTX) with folic acid taken at the second day after MTX. In case of insufficient response (DAS28-ESR ≥ 2.6) at the evaluation time points, the following per protocol treatment steps were advised: at week 8, MTX dosage was increased to 25 mg/week; at week 12, sulfasalazine (SSZ) 2000 mg/day was added; at week 20, SSZ dose was increased to 3000 mg/day. Tumor necrosis factor inhibitor (TNFi) was prescribed at week 24 for patients with persistent moderate disease activity (DAS28-ESR > 3.2). In case of sustained remission (DAS28-ESR < 2.6 for ≥ 6 months), medication was gradually reduced and eventually discontinued. In case of a disease flare (DAS28-ESR ≥ 2.6), the last effective medication or medication dose was restarted and treatment could be subsequently intensified. In patients with contraindications to specific medication, deviations from the protocol were allowed. Concomitant treatment with NSAIDs, prednisolone at a dosage of ≤ 10 mg/day and/or intra-articular corticosteroid injections were allowed at the discretion of the rheumatologist. Rheumatologists were free to diverge from the medication schedule at any time on clinical indication.

### Assessments

Serial measurements of disease activity and radiographic damage were performed according to a predefined follow-up scheme. Disease activity measures were collected at baseline and at every follow-up visit (weeks 8, 12, 20, 24, 36, and 52, and every 3 months thereafter) and consisted of the 28-tender joint count (TJC28), 28-swollen joint count (SJC28), ESR, CRP, and a patient rating for general health on a 100-mm visual analog scale (VAS-GH). Both the composite DAS28-ESR, which combines objective and subjective components of disease activity [18], and single CRP values were used as a measure of disease activity in all analyses to allow for better comparison with previous studies. Given the high correlation between composite DAS28-ESR and DAS28-CRP scores [22], the DAS28-CRP was not used. TJC28, SJC28, ESR, and VAS-GH were used to calculate the composite DAS28-ESR [18]. Total DAS28-ESR can be interpreted as low (≤ 3.2), moderate (> 3.2 to ≤ 5.1), or high (> 5.1) disease activity. A score < 2.6 corresponds to being in remission [19]. Although no validated cutoff criteria are available for CRP values in RA, values above 5 mg/l are generally considered indicative of the presence of inflammation and values > 30 mg/l for “active disease”. Based on clinical rules of thumb and cutoffs frequently used in previous research, CRP values were categorized as no inflammation (≤ 5), mild inflammation (> 5 to ≤ 10), moderate inflammation (> 10 to ≤ 30), and high inflammation (> 30) [23, 24].

Radiographs of the hand and feet were taken at baseline, 6 months, 12 months, 24 months, and 36 months and were consensus-scored by two trained readers using the modified Sharp/van der Heijde scoring (SHS) system [25]. Radiographs were scored in known (unblinded) chronological order [26, 27], allowing for progression only (i.e., no decrease in individual joint scores or “healing”). Relevant progression was defined as an increase of at least three units on the SHS for each of the four time intervals (0 to 6 months, 6 to 12 months, 12 to 24 months, 24 to 36 months), based on the calculated smallest detectable change (SDC) of 2.25 over the 5 repeated assessments using the ANOVA-based method described by Bruynesteyn et al. [28].

### Analysis

Time-integrated disease activity was calculated by the AUC method using trapezoidal integration [3]. In this method, the AUC between observations is the product of the time difference between the two measurements (in weeks) and the average of the two disease activity measurements. AUCs were calculated separately for each of the four time intervals of radiographic progression. In case of missing data on the disease activity parameter at a given time point, the area between the most recent non-missing time point and the next non-missing time point within a time interval was calculated (linear interpolation). No AUC for a time interval was calculated if the first or last time point was missing. Time-integrated disease activity scores per time interval were standardized by time (in weeks), yielding AUC values in the metric of the original measurement.

First, Pearson correlations between time-integrated disease activity and longitudinal radiographic progression (change from previous time point) at each of the follow-up time points were estimated. These correlations represent the inter-individual (between-person) associations between disease activity and outcome at the different time intervals. Next, individual trajectories of time-integrated disease activity scores at each of the four time intervals were plotted against cumulative radiographic progression scores. These plots visualize the intra-individual (within-person) relationship between time-integrated disease activity and radiographic progression over time. For each patient, the slopes of this relation for each time interval were calculated by dividing SHS progression (change from previous time point) by time-integrated disease activity in the respective time period. Pearson correlations between different time periods were determined to test the intra-individual associations between slopes of the relation between disease activity and outcome over time.

For each of the four follow-up time points (6, 12, 24, and 36 months), the proportion of patients showing relevant radiographic progression (≥ SDC of 3 on the SHS) in the preceding time interval was calculated and descriptively compared between patients with different predefined categories of time-integrated disease activity as measured with the DAS28-ESR and CRP using cross tabulations.

For all analyses, composite DAS28-ESR scores and CRP scores were used separately as indicator of disease activity. Primary analyses were done using available data. In the time-integrated AUC analyses, missing data on individual DAS28-ESR or CRP measurements within a time interval are linearly interpolated, if the missing value is not the first or last time point. If the first or last time point is missing, no AUC for this time interval is calculated. Missing radiographs at any of the time points result in no progression score for the patient in the preceding and subsequent time intervals. Patients with a missing radiograph at a certain time point were not censored from subsequent time periods. Missing data on radiographic assessments or other variables in the descriptive or correlational analyses were not imputed. For sensitivity analysis purposes, Pearson correlational analyses were performed on imputed data (multiple imputation; 10 imputations).

## Results

### Patient characteristics and outcomes

Out of 534 patients included in the 5 hospitals participating in the remission induction cohort, longitudinal radiographic data (at least 2 radiographic assessments) were available for 229 patients (42.9%) from 2 hospitals. The baseline characteristics of the patients are presented in Table 1. Most patients were female (63.3%) and the mean age was 57.5 years. All patients had active disease at baseline with a mean DAS28-ESR score of 4.9. Almost 47% already had at least one erosion at baseline. The majority of the patients were anti-cyclic citrullinated peptide antibody (anti-CCP) positive (58.8%) and rheumatoid factor (RF) positive (61.4%). Due to the observational nature of the sample, the number of missing values for patient-reported measures at baseline was generally higher than for clinical measures. A total of 191 (16.7%) out of the potential 1145 radiologic assessments were missing from baseline to 3-year follow-up (baseline 3.5%; 6 months 15.3%; 1st year 15.7%; 2nd year 25.3%; 3rd year 23.6%). Few patients had missing data on disease activity measurements at the different time points (0–14.0% for DAS28-ESR and 3.1–9.9% for CRP).

**Table 1:** Baseline characteristics of the patients (*N* = 229)

Characteristics	*n*	
Female, *n* (%)	229	145 (63.3)
Age, mean ± SD years	229	57.5 ± 15.0
BMI, mean ± SD kg/m^2^	220	26.4 ± 4.6
Symptom duration, median (IQR) weeks	228	13.0 (8.0–26.0)
RF positive, *n* (%)	228	140 (61.4)
Anti-CCP positive, *n*/total (%)	221	130 (58.8)
Fulfillment of ACR 1987 criteria, *n*/total (%)	225	178 (79.0)
Erosion ≥ 1 joint	226	102 (46.6)
DAS28-ESR, mean ± SD	229	4.9 ± 1.1
# of tender joints (28 assessed), median (IQR)	229	5 (2–10)
# of swollen joints (28 assessed), median (IQR)	229	8 (4–12)
ESR, median (IQR) mm/h	229	28.0 (16.0–42.0)
CRP, median (IQR) mg/l	229	12.0 (5.0–29.3)
VAS general health, median (IQR) 0–100 VAS	229	50 (30.0–66.5)
VAS pain, median (IQR) 0–100 VAS	229	50 (37.0–69.5)
VAS fatigue, median (IQR) 0–100 VAS	124	50 (24.3–71.5)
HAQ-DI, median (IQR)	191	1.1 (0.6–1.5)
SF-36 PCS, median (IQR)	199	35.4 (30.0–41.5)
SF-36 MCS, median (IQR)	199	47.9 (39.0–56.4)

*BMI* body mass index, *IQR* interquartile range, *RF* rheumatoid factor, *ACR* American College of Rheumatology, *DAS28* disease activity score based on 28-joint count, *ESR* erythrocyte sedimentation rate, *CRP* C-reactive protein, *VAS* visual analog scale, *HAQ* Health Assessment Questionnaire, *SF36* Short-Form 36 Health Survey, *PCS* physical component summary, *MCS* mental component summary

**Fig. 1:**
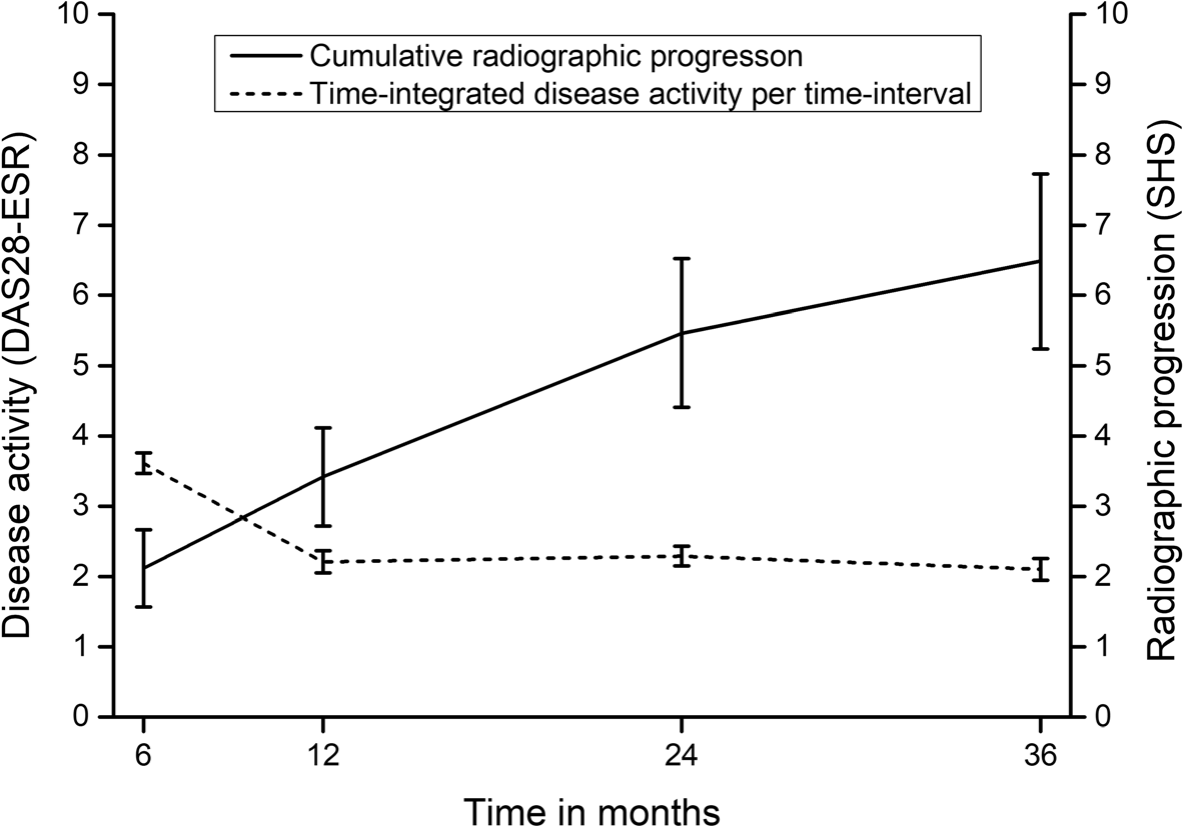
Mean standardized time-integrated DAS28 disease activity scores (from previous time point) versus mean cumulative SHS progression scores (from baseline). Error bars are 95% confidence intervals

Disease activity as measured with the DAS28-ESR decreased quickly from a mean (SD) of 4.92 (1.13, *n* = 229) at baseline to 2.83 (1.08, *n* = 225) after 6 months of T2T. DAS28-ESR scores further decreased to 2.50 (1.01, *n* = 220) after 1 year, 2.37 (0.98, *n* = 208) after 2 years, and 2.40 (0.98, *n* = 197) after 3 years of treatment. CRP scores showed a similar decrease over time, with mean scores decreasing from 18.6 (22.4, *n* = 222) at baseline to 7.5 (12.1, *n* = 226), 7.9 (10.8, *n* = 216), 8.4 (12.5, *n* = 210), and 8.2 (16.1, *n* = 200) after 6 months, 1 year, 2 years, and 3 years, respectively. Mean time-integrated DAS28-ESR scores in the four time intervals decreased from 3.64 (0.97, *n* = 229) in the first 6 months of treatment to 2.30 (1.04, *n* = 224) between 6 and 12 months, 2.37 (0.86, *n* = 217) between 1 and 2 years, and 2.13 (0.91, *n* = 206) between 2 and 3 years of treatment (Fig. 1). Mean time-integrated CRP scores were 9.62 (9.57, *n* = 228), 6.51 (6.01, *n* = 223), 7.40 (6.33, *n* = 215), and 7.45 (8.24, *n* = 207) at baseline–6 months, 6 months–1 year, 1–2 years, and 2–3 years, respectively.

Mean (SD) SHS radiographic damage at baseline was 4.68 (9.24). Radiographic joint damage kept increasing in the subsequent time intervals, with mean SHS (SD) progression scores of 2.11 (3.88, *n* = 193), 1.32 (1.88, *n* = 168), 1.85 (2.96, *n* = 150), and 1.43 (2.90, *n* = 148), respectively (Fig. 1). The number of patients with relevant progression (ΔSHS ≥ 3) in each of the time intervals was 48 (25.0%), 28 (17.2%), 35 (23.3%), and 26 (17.8%), respectively.

### Inter-individual relation between disease activity and outcome

At the group level, time-integrated DAS28-ESR scores were not significantly correlated with radiographic progression at the 6 month and the 2-year follow-up and only weakly at the 1-year and 3-year follow-up (Table 2). Inter-individual correlations were very similar for disease activity as measured with CRP, with time-integrated CRP and radiographic progression being significantly, but weakly, correlated at the 2-year and 3-year follow-up assessments only (Table 2). Similar correlations were found when using the imputed data. (Additional file 1: Table S1).

**Table 2:** Between-person Pearson correlations between standardized time-integrated (AUC) disease activity and radiological progression (from previous time point) for each time interval

	Baseline–6 months	6 months–1 year	1–2 years	2–3 years
DAS28-ESR	0.060 (*n* = 192)	0.170* (*n* = 164)	0.109 (*n* = 150)	0.209* (*n* = 146)
CRP	0.042 (*n* = 192)	0.137 (*n* = 166)	0.178* (*n* = 150)	0.275** (*n* = 147)

*P < 0.05; **P < 0.01. *n* = number of patients with an available time-integrated disease activity score and a radiographic progression score in the time interval

**Fig. 2:**
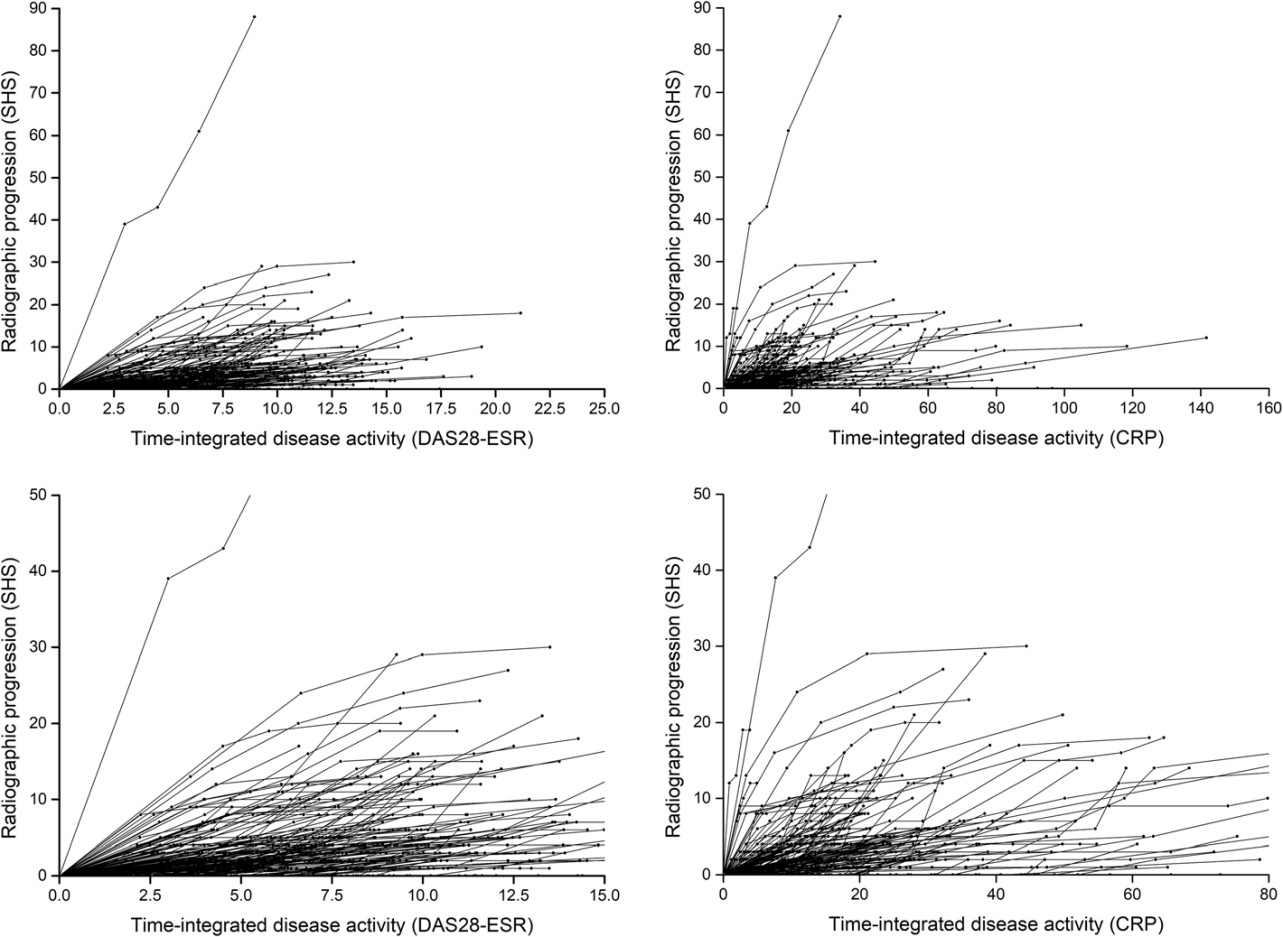
Intra-individual relations between time-integrated disease activity (standardized in weeks) and cumulative radiological progression during 3 years of follow-up (*n* = 229). Left column DAS28-ESR, right column CRP as disease activity indicator. Lower panel zoomed in for clarity. Every patient has its own line, composed of the 0–6-month, 6-month–1-year, 1-year–2-year, and 2-year–3-year values for time-integrated disease activity in relation to the cumulative SHS score at those time points

A wide inter-individual variation between disease activity and radiologic progression was also apparent from the individual trajectory plots (Fig. 2). Most patients showed limited radiographic progression over time. However, others showed more radiographic progression even at relatively low levels of time-integrated disease activity.

### Intra-individual relation between disease activity and outcome

In contrast to the absence of inter-individual correlation, however, within many individual patients, a fairly constant slope seemed to exist between exposure to disease activity and radiographic progression over time (Fig. 2). The slopes of the individual lines in each time interval (ΔSHS ÷ time-integrated disease activity) were significantly and substantially correlated over time for most time intervals (Table 3), confirming a relatively constant relation between disease activity and radiographic progression within patients. Especially, the slope of the first 6 months was moderately correlated with this slope at later time points, suggesting that, for individual patients, the first 6 months were indicative for the future relation between disease activity and outcome. In contrast to the DAS28-ESR, the slopes of CRP versus progression at the 2 to 3 years’ time interval were not significantly associated with those in previous time intervals, suggesting a change in the intra-individual association between CRP and progression at this point. Very similar, but slightly smaller, correlations were found when using the imputed data (Additional file 2: Table S2).

**Table 3:** Within-person Pearson correlations between slopes of time-integrated disease activity as measured by DAS28-ESR (top diagonal) and CRP (bottom diagonal) and radiographic progression over time

	Baseline–6 months	6 months–1 year	1–2 years	2–3 years
Baseline–6 months	–	0.394***	0.527***	0.592***
6 months–1 year	0.312***	–	0.172*	0.252**
1–2 years	0.643***	0.349***	–	0.478***
2–3 years	0.033	0.048	0.149	–

*P < 0.05; **P < 0.01; ***P < 0.001

### Post hoc group comparisons

Table 4 shows the proportion of patients experiencing radiographic progression larger than the smallest detectable change on the SHS from the preceding time segment stratified for patients with low, moderate, and high time-integrated DAS28-ESR scores in the same period. In total, 76 patients (33.2%) experienced relevant progression (ΔSHS ≥ 3) at least once across the 4 time intervals and 154 patients (67.2%) had at least one period of cumulative moderate or high disease activity. There was no significant relationship between experiencing relevant progression at least once across the 4 time intervals and moderate or high disease activity (independence chi-square test, *χ*^2^(1) = 0.40; *P* = 0.528).

**Table 4:** Proportion of patients experiencing radiographic progression (Δ SHS ≥ 3) within each time interval against categories of diseases activity (time-integrated standardized DAS28 scores) in the same period

Time-integrated disease activity	0–6-month progression		6-month–1-year progression		1-year–2-year progression		2-year–3-year progression	
	No	Yes	No	Yes	No	Yes	No	Yes
Low (DAS28 < 3.2)	58 (77.3%)	17 (22.7%)	113 (83.1%)	23 (16.9%)	100 (77.5%)	29 (22.5%)	111 (84.1%)	21 (15.9%)
Moderate (DAS28 3.2–5.1)	79 (73.8%)	28 (26.2%)	20 (83.3%)	4 (16.7%)	15 (75.0%)	5 (25.0%)	8 (61.5%)	5 (38.5%)
High (DAS28 > 5.1)	7 (70.0%)	3 (30.0%)	2 (66.7%)	1 (33.3%)	0 (0.0%)	1 (100%)	1 (100%)	0 (0.0%)
Total	144 (75.0%)	48 (25.0%)	135 (82.8%)	28 (17.2%)	115 (76.7%)	35 (23.3%)	120 (82.2%)	26 (17.8%)

A substantial proportion of patients with low (between 15.9 and 22.7%) or moderate (between 16.7 and 38.5%) cumulative disease activity experienced relevant radiographic progression at different time intervals. Similar proportions were found when using CRP as a measure of disease activity, with 12.0 to 22.6% of patients with no inflammation, 12.8 to 26.7% of patients with mild cumulative CRP scores, and 25.0 to 38.1% of patients with moderate CRP scores showing relevant radiographic progression across the different time intervals (Table 5).

**Table 5:** Proportion of patients experiencing radiographic progression (Δ SHS ≥ 3) within each time interval against categories of diseases activity (time-integrated standardized CRP scores) in the same period

Time-integrated inflammation	0–6-month progression		6-months–1-year progression		1-year–2-year progression		2-year–3-year progression	
	No	Yes	No	Yes	No	Yes	No	Yes
No (CRP ≤ 5)	65 (77.4%)	19 (22.6%)	87 (82.9%)	18 (17.1%)	71 (80.7%)	17 (19.3%)	81 (88.0%)	11 (12.0%)
Mild (CRP > 5 to ≤ 10)	49 (77.8%)	14 (22.2%)	34 (87.2%)	5 (12.8%)	31 (79.5%)	8 (20.5%)	22 (73.3%)	8 (26.7%)
Moderate (CRP > 10 to ≤ 30)	23 (67.6%)	11 (32.4%)	12 (75.0%)	4 (25.0%)	13 (61.9%)	8 (38.1%)	17 (73.9%)	6 (26.1%)
High (CRP > 30)	7 (63.6%)	4 (36.4%)	4 (80.0%)	1 (20.0%)	0 (0.0%)	2 (100%)	1 (50%)	1 (50.0%)
Total	144 (75.0%)	48 (25.0%)	135 (82.8%)	28 (17.2%)	115 (76.7%)	35 (23.3%)	121 (82.3%)	26 (17.7%)

## Discussion

In this retrospective study, we examined the longitudinal relationship between disease activity and radiographic joint damage in early RA patients following a strict T2T strategy in daily clinical practice. Despite a quick and sustained suppression of disease activity, mean radiographic damage still accumulated during follow-up and a substantial number of patients with low or moderate disease activity experienced relevant radiographic progression. Time-integrated disease activity and radiographic progression were not or only weakly correlated at the group level in the first 3 years of treatment. In contrast, within individual patients, slopes of the relation between disease activity and radiographic progression were significantly and moderately correlated over time for most time intervals. Moreover, the relation between disease activity and radiographic progression in the first 6 months of treatment was already predictive for this relation at later time intervals. Taken together, these findings indicate that, in the current intensive T2T paradigm in early RA, joint destruction is mainly driven by factors other than consistent high disease activity and instead appears to be an individually determined disease process.

The overall radiographic progression in our study was substantially lower in comparison with studies from the last decades [29, 30]. This improvement is most likely due to the more intensive treatment strategy, such as more intensive use of csDMARDs and the availability of biological DMARDs. Previously, we showed that the implementation of T2T in the current cohort led to more rapid and higher DAS28-ESR remission rates after 1 year than usual care [31]. Even in the long term, suppression of disease activity resulted in limited radiographic damage at the group level [16]. However, some patients still continue to experience joint destruction, even when achieving relatively low disease activity. Two other longitudinal cohort studies also demonstrated that substantial proportions of patients (7 to 17%) in sustained remission still had relevant progression of joint damage and 15 to 20% developed erosions in a previously unaffected joints [32, 33].

Previous studies in conventionally treated patients have generally demonstrated clear associations between the cumulative amount of disease activity (as measured by time-integration of laboratory-based disease activity parameters) and radiographic progression [5, 8, 9]. However, even before the advent of T2T, the absolute strength of the reported correlations already showed a tendency to decrease over time with increasingly intensive and earlier use of conventional DMARDs. For example, Van Leeuwen et al. [5] reported a strong correlation of 0.64 between time-integrated CRP values and radiological progression over 3 years of follow-up in established RA patients treated with NSAIDs and low-dose conventional DMARDs. Plant et al. [8] demonstrated a correlation of 0.50 between time-integrated CRP level and change in Larsen score after 5 years of follow-up in active RA patients treated with conventional DMARDs. Knijff et al. [9] reported weaker correlations of 0.38 and 0.25 between radiographic damage and time-integrated CRP and rheumatoid factor, respectively, but a fairly constant slope between exposure to disease activity over time and radiographic progression in individuals. In a later study, Wick et al. [11] initially also found a weak correlation (*r* = 0.31) at the group level between time-integrated CRP and joint damage over 2 years of follow-up in RA patients starting treatment with DMARDs within 2 months after diagnosis. After adding an individual factor for each patient to the model, however, a strong and highly significant correlation (*r* = 0.58) emerged.

Our study confirms these results by showing a very weak group-level correlation between time-integrated disease activity and radiographic progression over time in RA patients treated with a modern T2T treatment strategy. It is also in line with a recent study that showed that the proportion of variance in radiographic progression explained by cumulative measures of disease activity clearly decreased with increasingly aggressive csDMARD strategies in daily practice [14]. However, this study only examined associations between cumulative disease activity and 5-year radiographic progression at the group level and did not examine this association within individual patients over time. The current study extends the findings of Wick et al. [14] showing that, although between-person associations may be negligible, within individual patients a fairly constant and moderate relationship exists between disease activity and radiographic progression over time with a relevant number of patients with low or moderate disease activity experiencing radiographic progression.

Clinical trials of biological DMARDs in established RA patients after previous csDMARD failure also showed a subsequent disconnect between disease activity and radiographic progression [12, 13]. As such, the current study adds to the body of evidence that suggests that in the current paradigm of earlier and more intensive treatment, joint damage is no longer clearly a direct result of high disease activity for all patients. Instead, radiographic progression appears to be an increasingly individual process, driven by factors other than consistent high disease activity as measured by CRP or composite indices such as the DAS28-ESR.

The finding that for most patients the individual slope of disease activity as measured with the DAS28-ESR and progression seems to be reasonably linear is relevant for the treatment of patients in daily clinical practice, aiming for individualized targets/medicines. The majority of the patients receiving T2T demonstrated little radiographic progression. However, a considerable number of patients with consistently moderate, or even low, disease activity still developed radiographic progression. As the individual slope in the first 6 months of treatment was moderately associated with this slope in the longer term, patients at risk of joint damage might be identifiable early in the disease process. This suggests that radiographic evaluation at both treatment initiation and after 6 months of treatment might be valuable, and treatment optimization might be needed even when patients have low disease activity. Treatment decisions in early phase of RA may thus need to be based on consideration of disease activity as well as radiographic progression. For patients with radiographic progression, rheumatologists could consider initiating biological or targeted synthetic treatment, even if disease activity alone would not merit such a therapeutic change.

Besides early progression, assessing other markers of disease activity may also have added value in identifying patients at risk. For instance, a recent study in early RA patients showed that repeated measures of interleukin-6 levels were associated with structural damage independently from the DAS28 [34].

Although RF and anti-CCP seropositivity was rather low in the current sample and 21% of the patients did not fulfill the ACR 1987 criteria for RA at baseline, this is not uncommon in recent onset arthritis patients. For instance, only 83% of the very early RA patients in the cohort study by Kaarela et al. fulfilled the ACR RA criteria [35]. In addition, trials that included early RA patients according to the ACR 1987 criteria still showed proportions of positive auto-antibodies close to those in the current sample. For example, the COBRA-light study reported 62% anti-CCP positivity and 58% RF positivity and the BeSt study reported 62% anti-CCP positivity and 66% RF positivity [36, 37]. The value of the ACR 1987 criteria (or the number of criteria that need to be met) in early RA has been criticized [35], and since the start of the current cohort in 2006, new criteria have been published in 2010 that are more sensitive in early disease [38].

A strength of this study is the real-life setting which makes the results more generalizable to daily clinical practice. Despite the interesting results, however, it should be noted that the study has some limitations that may have affected the results. The retrospective observational design of this study does not allow any causal inference about the association between disease activity and radiographic outcome. Also, given the limited sample size, statistical analyses and group comparisons were not adjusted for or stratified by baseline variables that may be associated with disease activity and radiographic damage such as baseline erosive disease and BMI. Additionally, the real-world nature of the study may have introduced biases, including confounding by indication. Also, there were a substantial number of patients with missing values on radiographic progression, especially at the 2- and 3-year follow-up assessments, which may have biased the results. These missing values may have been not missing at random, as it is conceivable that radiographs were more frequently not taken in patients with adequately controlled disease. The findings should also be interpreted in the light of the specific treatment protocol used, with step-up treatment of adding a second csDMARD first and only anti-TNFs as a biological treatment option. For instance, new treatment options with different modes of action have recently emerged, such as JAK inhibitors. Finally, as almost all patients had low or moderate disease activity at the different follow-up intervals, caution should be taken in extrapolating the findings concerning the association between disease activity and radiographic progression to patients with high disease activity.

## Conclusions

In conclusion, this study shows a disconnection between time-integrated disease activity and radiographic progression at the group level in real-world patients treated according to a strict T2T protocol. Radiographic progression seems to be an individual process in these patients, determined by factors other than currently used disease activity indicators. Even though T2T results in acceptable control of disease activity for most patients, individual patients with low or moderate disease activity may still be at risk of longer-term radiographic damage.

## Supplementary information

**Additional file 1:** **Table S1.** Pooled between-person Pearson correlations between standardized time-integrated (AUC) disease activity and radiological progression (from previous time point) for each time interval based on multiple imputation (10 imputations).

**Additional file 2.** **Table S2.** Pooled within-person Pearson correlations between time-integrated disease activity as measured by DAS28-ESR (top diagonal) and CRP (bottom diagonal) and radiographic progression over time based on multiple imputation (10 imputations).

## Data Availability

The datasets used and/or analyzed during the current study are not publicly available due to legal restrictions related to data privacy protection. However, the data are available on reasonable request after authorization of the DREAM board. Researchers interested in data access may contact the DREAM board via http://www.dreamregistry.nl/en.

## Abbreviations

ANOVA: Analysis of variance
anti-CCP: Anti-cyclic citrullinated peptide antibody
AUC: Area under the curve
CRP: C-reactive protein
csDMARDs: Conventional synthetic disease-modifying antirheumatic drugs
DAS28-ESR: 28-joint disease activity score-erythrocyte sedimentation rate
DMARDs: Disease-modifying antirheumatic drugs
DREAM: Dutch Rheumatoid Arthritis Monitoring
ESR: Erythrocyte sedimentation rate
MTX: Methotrexate
NSAIDs: Non-steroidal anti-inflammatory drugs
RA: Rheumatoid arthritis
RF: Rheumatoid factor
SDC: Smallest detectable change
SHS: Sharp/van der Heijde scoring
SJC: Swollen joint count
SSZ: Sulfasalazine
T2T: Treat-to-target
TJC: Tender joint count
TNFi: Tumor necrosis factor inhibitor
VAS-GH: Visual analog scale for general health
