# Supplementary material for: Radiographic progression can still occur in individual patients with low or moderate disease activity in the current treat-to-target paradigm: real-world data from the Dutch Rheumatoid Arthritis Monitoring (DREAM) registry
Source: Arthritis Res Ther. 2019 Nov 12;21:237. doi: 10.1186/s13075-019-2030-8 (PMC6852758; doi:10.1186/s13075-019-2030-8)
Supplement: Supplementary file 1 — Additional file 1: Table S1. Pooled between-person Pearson correlations between standardized time-integrated (AUC) disease activity and radiological progression (from previous time point) for each time interval based on multiple imputation (10 imputations). [file 13075_2019_2030_MOESM1_ESM.docx]

Table S1: Pooled between-person Pearson correlations between standardized time-integrated (AUC) disease activity and radiological progression (from previous time point) for each time interval based on multiple imputation (10 imputations).

|  | baseline–6 months  (n=229) | 6 months–1 year  (n=229) | 1–2 year  (n=229) | 2–3 year  (n=229) |
| --- | --- | --- | --- | --- |
| DAS28-ESR | 0.171* | 0.019 | 0.055 | 0.228** |
| CRP | 0.073 | 0.053 | 0.131 | 0.328*** |

* P<0.05; ** P<0.01. n = number of patients with an available time-integrated disease activity score and a radiographic progression score in the time interval.
