# Supplementary material for: Radiographic progression can still occur in individual patients with low or moderate disease activity in the current treat-to-target paradigm: real-world data from the Dutch Rheumatoid Arthritis Monitoring (DREAM) registry
Source: Arthritis Res Ther. 2019 Nov 12;21:237. doi: 10.1186/s13075-019-2030-8 (PMC6852758; doi:10.1186/s13075-019-2030-8)
Supplement: Supplementary file 2 — Additional file 2. Table S2. Pooled within-person Pearson correlations between time-integrated disease activity as measured by DAS28-ESR (top diagonal) and CRP (bottom diagonal) and radiographic progression over time based on multiple imputation (10 imputations). [file 13075_2019_2030_MOESM2_ESM.docx]

Table S2: Pooled within-person Pearson correlations between time-integrated disease activity as measured by DAS28-ESR (top diagonal) and CRP (bottom diagonal) and radiographic progression over time based on multiple imputation (10 imputations)..

|  | baseline–6 months | 6 months–1 year | 1–2 year | 2–3 year |
| --- | --- | --- | --- | --- |
| baseline–6 months | – | 0.378*** | 0.474*** | 0.516*** |
| 6 months–1 year | 0.298*** | – | 0.112 | 0.170 |
| 1–2 year | 0.579*** | 0.295*** | – | 0.419*** |
| 2–3 year | 0.045 | 0.049 | 0.166 | – |

*** P<0.001.
